# Asymptomatic Intraosseous Meningioma of the Humerus: A Case Report and Review of the Literature

**DOI:** 10.7759/cureus.32590

**Published:** 2022-12-16

**Authors:** Shivam H Patel, Kelly N McKnight, Parth A Vyas

**Affiliations:** 1 School of Medicine and Health Sciences, University of North Dakota, Grand Forks, USA; 2 Orthopaedic Surgery, Sanford Health, Fargo, USA

**Keywords:** metastasis, pathology, surgery, humerus, meningioma, extradural, intraosseous

## Abstract

Meningiomas are the most common central nervous system tumor. They are typically benign neoplasms but may produce neurological symptoms due to mass effect. Meningiomas may also extend to extradural locations; however, these account for only a small percentage of all meningiomas. Most extradural meningiomas arise in intraosseous locations, usually within the cranial bones or vertebrae. However, this is a rare case of extradural extension of an asymptomatic intracranial meningioma to the proximal humerus in the absence of any musculoskeletal symptoms. To the best of our knowledge, this presentation of an extradural intraosseous meningioma has not previously been reported in the literature. We present a case of an incidental intraosseous meningioma in a 66-year-old man. This patient was initially being screened for metastasis of stage IA1 adenocarcinoma of the lung, and a positron emission tomography (PET) scan revealed a focus of activity in the proximal diaphysis of the right humerus suspicious for malignancy. The upper extremity magnetic resonance imaging (MRI) demonstrated an indeterminate lesion. Curettage of the humeral lesion revealed an intraosseous psammomatous meningioma without evidence of metastatic lung carcinoma. Our case report aims to illustrate the importance of considering alternative metastatic sources, such as intracranial meningioma, during the investigation of an indeterminate bony lesion. This is the first case to illustrate asymptomatic intraosseous meningioma in an appendicular skeletal location, highlighting the need for thorough source investigation.

## Introduction

Meningiomas are the most common tumor of the central nervous system (CNS) and originate from the arachnoid cells of the meningeal layers covering the brain and spinal cord [1]. Meningiomas account for an estimated 34%-37% of all primary CNS tumors, and although they are typically benign, meningiomas may produce neurological symptoms and deficits in some patients [2-4]. Rarely, meningiomas may arise as primary tumors in extradural locations or as extracranial lesions secondary to metastasis [5]. Extradural meningiomas account for only 1%-2% of all meningiomas with a large proportion of these found in intraosseous locations [5]. Of the intraosseous meningiomas, most are located within cranial bones or vertebrae [5].

Contrasting to meningiomas of the axial skeleton, intraosseous meningiomas of the appendicular skeleton are reported with much less frequency. To our knowledge, there are only a sparse number of reports of intraosseous meningiomas within the appendicular skeleton [6-8]. Additionally, intraosseous meningiomas generally present with pain and swelling leading to a wide differential diagnosis, including both primary and secondary bone cancers, which warrant further clinical investigation [6,8,9]. To the best of our knowledge, this is the first case of an asymptomatic extracranial intraosseous meningioma in an appendicular skeletal location. We present the case of a 66-year-old male who was incidentally found to have a painless and asymptomatic bony lesion of the humerus during screening for carcinoma of the lung, which was later identified as a meningioma.

## Case presentation

Case report

**Figure 1:**
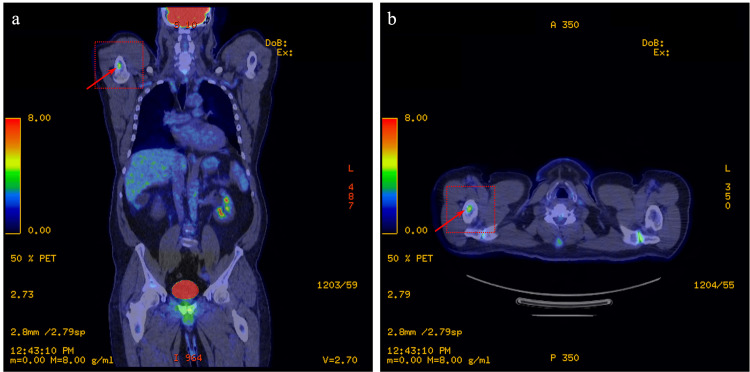
Positron emission tomography (PET). (a) Coronal and (b) axial positron emission tomography imaging demonstrating increased uptake in the right proximal humerus. Areas of interest are depicted by red arrow.

**Figure 2:**
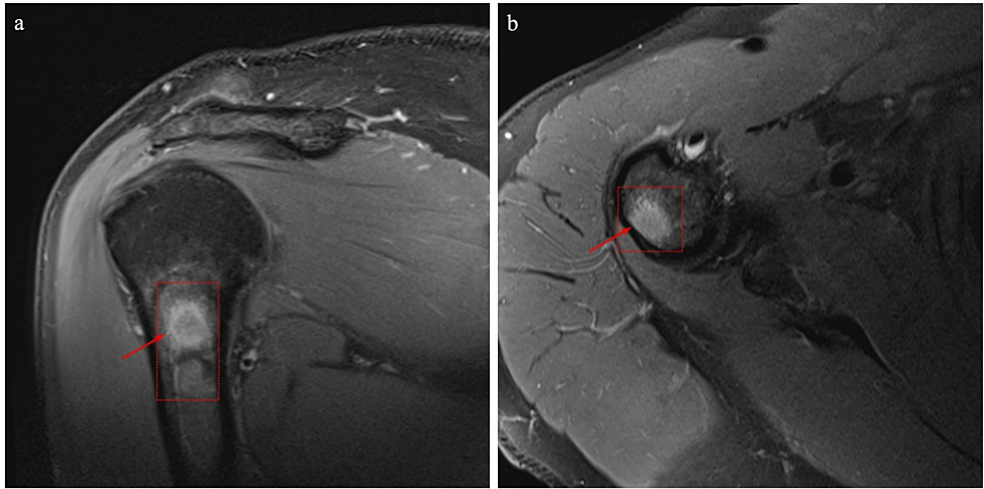
Right shoulder magnetic resonance imaging. (a) Coronal and (b) axial proton density magnetic resonance images demonstrating a bone lesion concerning for metastatic disease of the right humerus at the level of the surgical neck and extending into the proximal humeral shaft. Areas of interest are depicted by red arrow.

**Figure 3:**
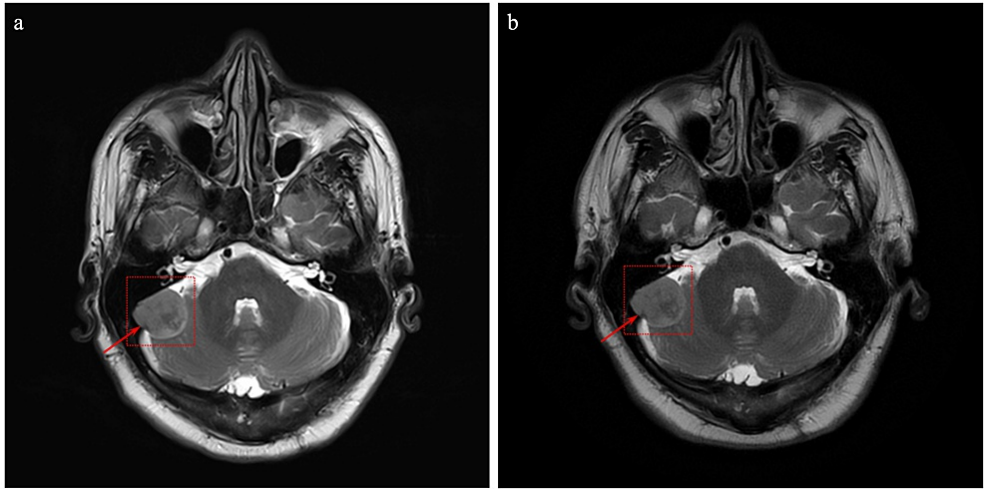
Brain magnetic resonance imaging. Imaging demonstrating a stable 2.4 cm meningioma along the right cerebellopontine angle at (a) initial discovery and (b) four months following initial magnetic resonance imaging. Areas of interest are depicted by red arrow.

The patient, a 66-year-old male, was scheduled for a positron emission tomography (PET) scan following a continued interval increase in the size of a solid irregular lung nodule seen on computerized tomography (CT) measuring 9 mm x 8 mm in the right upper lobe. Biopsy of the pulmonary nodule revealed stage IA1 adenocarcinoma with strong expression of thyroid transcription factor-1 (TTF-1), supporting lung origin. At the time of the PET scan, a suspicious focus of activity was noted in the proximal diaphysis of the right humerus (Figures 1a, 1b). Right shoulder magnetic resonance imaging (MRI) with and without contrast was ordered to assess for possible metastatic disease. MRI revealed a bone lesion concerning for metastatic disease of the right humerus at the level of the surgical neck and extending into the proximal humeral shaft (Figures 2a, 2b). The lesion appeared distinct from typical enchondroma on MRI as it was somewhat irregular in appearance on the T2-weighted sequence and internal foci suggesting that the chondroid matrix was not appreciated. As no lymph node involvement was noted on the PET scan, it was felt that the lesion represented another type of malignancy, rather than metastatic lung cancer. An MRI of the brain was also ordered to evaluate for the presence of lesions which demonstrated a 2.4 cm meningioma along the right cerebellopontine angle (Figure 3a). A repeat brain MRI four months following the initial MRI showed that the meningioma was stable, and the patient remained asymptomatic from a neurological standpoint (Figure 3b).

**Figure 4:**
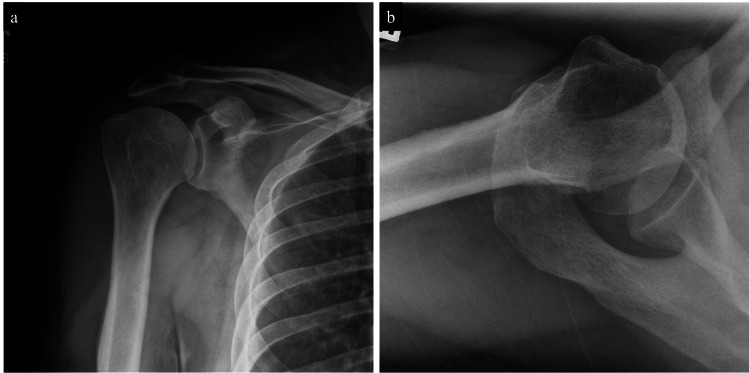
Preoperative shoulder radiographs. (a) Anteroposterior and (b) axial preoperative shoulder radiographs demonstrating no apparent abnormality.

The patient was referred to orthopedic surgery for further evaluation and biopsy of the indeterminate humeral lesion. On initial presentation to the orthopedic oncology clinic, it was noted that the patient had a history of adenocarcinoma of the right lung, seropositive rheumatoid arthritis, hyperlipidemia, and obesity. The patient also had a remote history of smoking, less than 10-pack-years. His physical exam was unremarkable. Notably, an examination of bilateral shoulders revealed a full range of movement without pain. There was no evidence of tenderness overlying the region of the proximal humerus, nor was there evidence of distal neurovascular deficit. No apparent abnormality was initially noted on the x-ray (Figures 4a, 4b). However, given the indeterminate nature of the lesion on MRI (Figure 2), a decision between the provider and patient was made to proceed with a biopsy to rule out malignancy or metastatic disease. The lesion was not found feasible for needle biopsy by interventional radiology, and therefore, an open biopsy was recommended.

The patient underwent surgery to obtain an open biopsy specimen for pathological analysis by curettage under general endotracheal anesthesia. Ancef was used as preoperative antibiotic prophylaxis, and a formal timeout was conducted. An incision was made on the anterior humerus in line with the deltopectoral interval. The basilar vein was retracted with the deltoid, and the proximal humerus was exposed. The lesion was confirmed with fluoroscopy, and an oblong window in the bone was created with bur and osteotomes. The underlying tissue was curetted and sent for intraoperative frozen section consultation. The sample appeared to be adequate; however, a definitive diagnosis was difficult to obtain immediately. A thorough curettage was performed and submitted to pathology. The entire cavity was treated with phenol for three minutes, followed by alcohol. This cycle was repeated three times, followed by thorough irrigation. HydroSet was mixed, and 10 mL was injected inside the cavity. Final x-rays were obtained. Once HydroSet was adequately set, the bone window was cleaned with a curette and infiltrated with phenol and alcohol, and it was replaced back on the outer cortex using a tamp. Closure was completed in standard fashion; sterile dressing was applied; and the patient was shifted out of the operating room.

**Figure 5:**
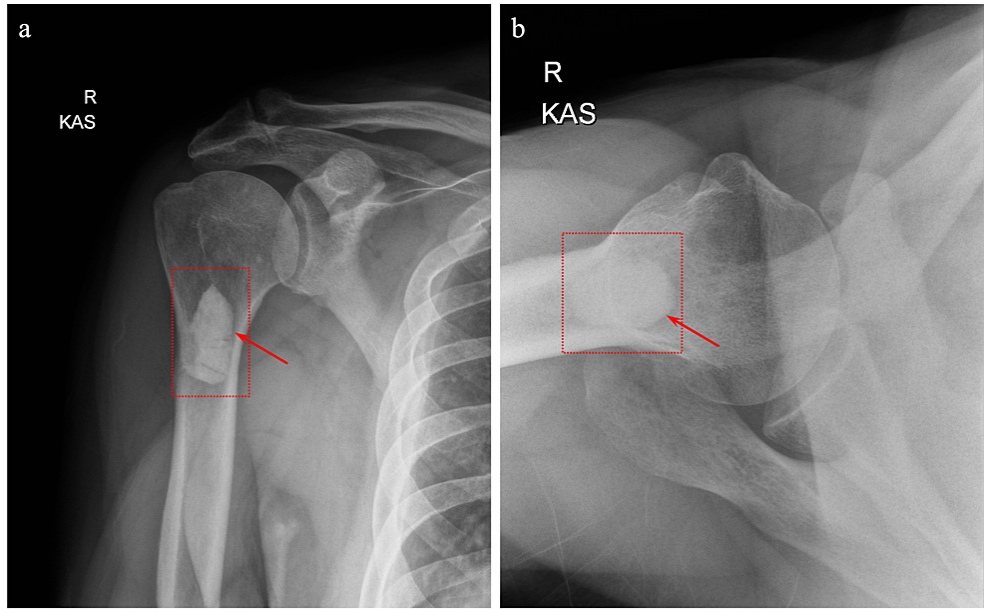
Postoperative shoulder radiographs at four months. (a) Anteroposterior and (b) axial radiographs reveal bony remodeling after surgery and good position of the bone graft substitute. Areas of interest are depicted by red arrow.

Postoperatively, the patient was allowed the motion of the shoulder and use of the arm as tolerated with a lifting restriction of 15 pounds. He was seen for postoperative follow-up where imaging revealed bony remodeling after surgery and good position of the bone graft substitute (Figures 5a, 5b).

Tissue pathology

**Figure 6:**
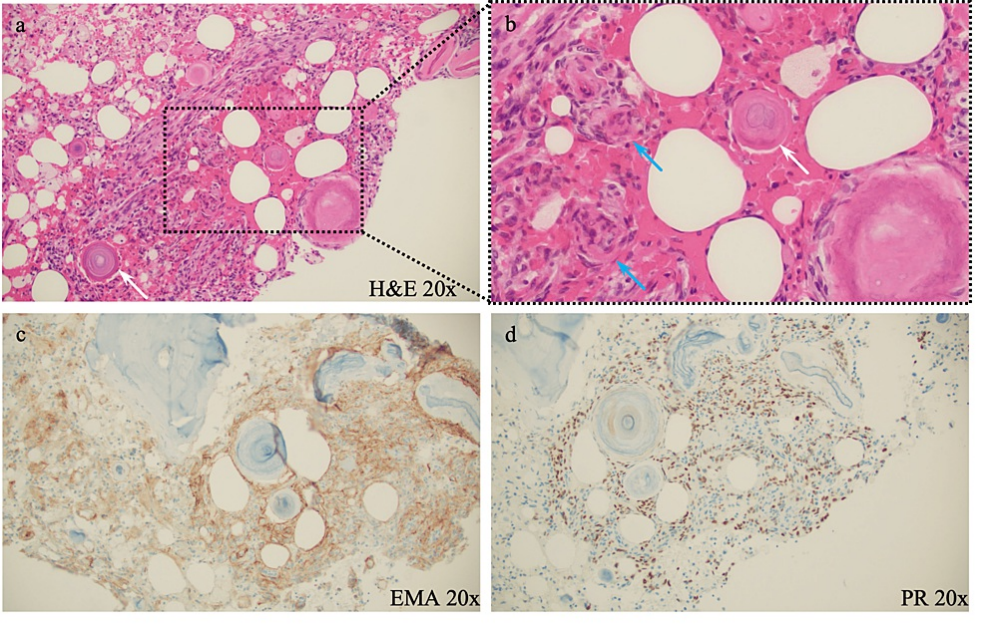
Pathology slides from curettage demonstrate an intraosseous psammomatous meningioma. (a) Hematoxylin and eosin (H&E) 20x and (b) magnified field of view from H&E 20x panel (black dashed line). H&E staining displays plump spindle and polyhedral cells with oval to elongate dark staining nuclei focally arranged in whorls, exampled by blue arrows. Numerous focally calcified psammoma bodies can also be seen throughout the sample, exampled by white arrows. (c) Epithelial membrane antigen (EMA) 20x is shown to be diffusely positive, demonstrated by brown staining throughout the sample. (d) Progesterone receptor (PR) 20x is shown to be diffusely positive, demonstrated by brown staining throughout the sample.

A curettage (2 cm x 2 cm x 0.6 cm aggregate) of bony tissue was submitted to pathology in formalin. Sections of the mass demonstrated an intraosseous psammomatous meningioma without evidence of metastatic carcinoma. Plump spindle and polyhedral cells with oval to elongate dark staining nuclei focally arranged in whorls were revealed within the mass (Figures 6a, 6b). Numerous focally calcified psammoma bodies separated by foamy macrophages and areas of woven bone formation were also noted (Figures 6a, 6b). Foamy macrophages were confirmed with positive immunohistochemistry for cluster of differentiation 68 (CD68). The tumor cells were diffusely positive for epithelial membrane antigen (EMA) (Figure 6c), and many cells were stained with progesterone receptor (PR) (Figure 6d). The proliferation rate as estimated from the Ki-67 nuclear labeling index was less than 1%, and no significant cytological atypia or mitotic activity was noted. Staining was negative for anti-cytokeratin (CAM5.2), family of calcium-binding proteins (S100), somatostatin receptors type 2 (SSTR2), desmin, alpha-smooth muscle actin (SMA), and special AT-rich sequence-binding protein-2 (SATB2). The morphological features and immunochemical findings supported the diagnosis of extracranial intraosseous meningioma. Metastasis of adenocarcinoma from the lung was ruled out.

## Discussion

Meningiomas are benign slow-growing tumors originating from meningothelial cells and are the most frequently occurring primary tumor of the CNS, accounting for 34%-37% of all primary intracranial tumors [2,10-12]. On rare occasion, meningiomas may arise in extradural locations, such as skin, lungs, and intraosseous locations [13]. Extradural meningiomas account for only a small percentage (1%-2%) of all meningiomas and may arise as a primary extradural meningioma or secondary to a metastatic meningioma of the brain [5,14-16]. Among the extracranial meningiomas, intraosseous meningiomas account for the majority and are reported most commonly at sites within the axial skeleton, such as the bones of the skull and vertebrae [14]. In fact, an estimated 70% of extradural meningiomas are found to involve bones of the skull [7]. Case reports of intraosseous meningiomas indicate that patients present with musculoskeletal symptoms such as pain, swelling, site tenderness, and paresthesia that warrant further investigation [9,17]. Several reports now exist of intraosseous meningiomas within the axial skeleton; however, reports of meningiomas of the appendicular skeleton are particularly infrequent. Of the limited case reports on appendicular intraosseous meningiomas, all patients presented with pain or tenderness to palpation in the region overlying the meningioma [6,7]. Provided the rarity of this problem, patients are not routinely screened for extradural meningiomas unless presenting with symptomatic complaints, and thus, painless intraosseous meningiomas are less likely to be discovered or reported [16]. We report a very rare case of asymptomatic intraosseous meningioma in an appendicular skeletal location.

In this case, incidental discovery of the humeral intraosseous meningioma was secondary to screening for carcinoma of the lung via PET scan which revealed fluorodeoxyglucose (FDG) uptake at the proximal humerus (Figure 1). A follow-up MRI of the upper extremity demonstrated an indeterminate lesion (Figure 2). The incidental discovery of the proximal humerus lesion via PET and MRI prompted an MRI scan to screen for the presence of brain lesions which revealed a 2.4 cm meningioma at the right cerebellopontine angle (Figure 3a). While it is challenging to determine the temporal characteristics of the intracranial and extradural intraosseous meningiomas, it is more likely that the humeral lesion occurred as a distant metastasis of the intracranial meningioma.

Distant metastasis is most commonly a feature of high-grade meningiomas (World Health Organization (WHO) grade 3) and is estimated to occur in only ~0.76% of all cases [18]. Metastasis is much more likely to occur in WHO grade 3 meningiomas, and meningioma reoccurrence also increases in tumors that demonstrate a Ki-67 proliferation index of greater than 4% [3,7,18]. Pathological analysis of the humeral meningioma in this case demonstrated a proliferative index of <1%. The lack of high mitotic activity and atypical features on histology suggest that the intracranial meningioma in this report may not be WHO grade 3 anaplastic. Additionally, the curettage samples were diffusely positive for PR (Figure 6d) which further suggests benign status [19]. While PR staining alone cannot be used to predict the prognosis of meningiomas, WHO grades 2 and 3 meningiomas are more likely to have fewer receptors [19]. PR status when used in conjunction with proliferative index data can however be used to predict meningioma prognosis [19]. The intracranial meningioma in this case is assumed to be benign based on no interval increase in size, rather than histologically determined benign status as there is no indication for resection at this time. The presence of distant metastasis in this case of benign intracranial meningioma is rare but also demonstrates a challenge in the true estimation of extracranial metastasis.

It is estimated that up to 56.2% of metastatic meningiomas originate from benign meningiomas and that 31.3% of metastatic meningiomas are found incidentally [20]. This statistic further highlights the challenge in estimating the true prevalence of extradural meningiomas in patients who present with no clinical neurological or musculoskeletal complaints, such as the patient in the present case. Rather than suggesting implementation of whole-body screening, we demonstrate why it may be prudent to include primary or metastatic intraosseous meningioma in the differential diagnosis of indeterminate lesions suspicious for bone cancer which present either symptomatically or are found incidentally. Further, histological assessment should always be considered as intraosseous meningiomas are unable to be diagnosed by imaging alone due to rarity and non-specific radiologic findings [7].

## Conclusions

At the time of writing this report, a review of the literature revealed no cases of clinically asymptomatic intraosseous meningiomas of the appendicular skeleton. In the present case, the patient was asymptomatic in that there was no pain or tenderness in the region overlying the proximal humerus, no abnormalities with shoulder range of motion, and no neurovascular deficits. Further, no evidence of focal neurological complaints was present, and thus, suspicion for an intraosseous meningioma was low until a screening MRI of the brain revealed an intracranial meningioma at the right cerebellopontine angle. Interestingly, metastatic meningiomas are reported to be clinically silent in ~31% of cases. However, accurate estimates of the frequency of clinically silent intraosseous meningiomas are not available in the literature. This does not exclude the fact that they may occur in similar frequency as painful appendicular intraosseous meningiomas, but rather that the number of painless metastatic intraosseous meningiomas may be underreported due to the lack of screening in the absence of symptoms. While there are no formal recommendations for routine screening following meningioma resection, providers may consider a surveillance protocol for patients who have WHO grade 3 meningiomas or tumors with characteristic high mitotic activity as determined by the Ki-67 proliferation index. Further, as lower-grade meningiomas are also known to metastasize, it would be warranted to include a diagnosis of intraosseous meningiomas when investigating indeterminate bony lesions.

To the best of our knowledge, this is the first report of an asymptomatic intraosseous meningioma in a location of the appendicular skeleton. Although metastatic meningioma to intraosseous locations is reported to be rare, its actual incidence is difficult to quantify due to an unknown number of asymptomatic patients.
